# An Ultra-High Discrimination Y Chromosome Short Tandem Repeat Multiplex DNA Typing System

**DOI:** 10.1371/journal.pone.0000688

**Published:** 2007-08-01

**Authors:** Erin K. Hanson, Jack Ballantyne

**Affiliations:** 1 Graduate Program in Biomolecular Science, University of Central Florida, Orlando, Florida, United States of America; 2 Department of Chemistry, University of Central Florida, Orlando, Florida, United States of America; 3 National Center for Forensic Science, Orlando, Florida, United States of America; Texas A&M University, United States of America

## Abstract

In forensic casework, Y chromosome short tandem repeat markers (Y-STRs) are often used to identify a male donor DNA profile in the presence of excess quantities of female DNA, such as is found in many sexual assault investigations. Commercially available Y-STR multiplexes incorporating 12–17 loci are currently used in forensic casework (Promega's PowerPlex® Y and Applied Biosystems' AmpF*l*STR® Yfiler®). Despite the robustness of these commercial multiplex Y-STR systems and the ability to discriminate two male individuals in most cases, the coincidence match probabilities between unrelated males are modest compared with the standard set of autosomal STR markers. Hence there is still a need to develop new multiplex systems to supplement these for those cases where additional discriminatory power is desired or where there is a coincidental Y-STR match between potential male participants. Over 400 Y-STR loci have been identified on the Y chromosome. While these have the potential to increase the discrimination potential afforded by the commercially available kits, many have not been well characterized. In the present work, 91 loci were tested for their relative ability to increase the discrimination potential of the commonly used ‘core’ Y-STR loci. The result of this extensive evaluation was the development of an ultra high discrimination (UHD) multiplex DNA typing system that allows for the robust co-amplification of 14 non-core Y-STR loci. Population studies with a mixed African American and American Caucasian sample set (n = 572) indicated that the overall discriminatory potential of the UHD multiplex was superior to all commercial kits tested. The combined use of the UHD multiplex and the Applied Biosystems' AmpF*l*STR® Yfiler® kit resulted in 100% discrimination of all individuals within the sample set, which presages its potential to maximally augment currently available forensic casework markers. It could also find applications in human evolutionary genetics and genetic genealogy.

## Introduction

The unique biology of the Y chromosome has led to the widespread use in forensic and evolutionary studies of genetic markers thereon in determining patrilineal relationships within and between population groups [1]–[6]. A subset of these markers, minisatellites or short tandem repeats (Y-STRs), are now used routinely in certain forensic casework situations [7]–[18]. Their intended use is not to supplant the current battery of autosomal STR loci but to apply them to certain defined casework situations whereby the traditional autosomal loci would not be expected to yield sufficient probative information. Y-STRs are particularly beneficial for the analysis of the male donor in a male/female DNA admixture when the female DNA component is present in vast excess (e.g. ≥100×) and when traditional autosomal STR analysis fails or is not possible [19]–[22].

Autosomal STR analysis may not be possible if the sample contains an admixture of body fluids other than semen, such as in saliva/saliva mixtures, saliva/vaginal secretion mixtures, or fingernail scrapings comprising cells from the (female) victim and cells from the perpetrator. In these types of samples a differential extraction to separate the male and female cells is not possible with current technology and the male component is often not detectable with the autosomal STR multiplex systems routinely used due to a titration of critical PCR reagents by the major contributor in the sample [22]. Autosomal STR analysis may also fail with some semen containing samples in which the sperm are present in very low copy number, or are present in an extremely fragile state, such as in extended interval (i.e. >48 h) post-coital samples. Differential extraction of these particular samples may yield no profile from the male donor due to a combination of premature lysis of the sperm's cellular constituents into the non-sperm fraction and to sperm loss during the physical manipulations required of the DNA isolation process. Therefore, the use of Y-STRs, which target only the male fraction, eliminate the need for a differential extraction process and lessen the potential to lose the trace amounts of male DNA that may be present.

There are several additional benefits of Y-STR analysis in forensic casework. Y-STR analysis allows for the facile determination of the number of male contributors in mixtures. Y-STR profiles are hemizygous in nature, with one allele being found at most loci (the exception being a small number of multi-copy loci). Multiple alleles at single copy loci give a clear indication of the number of male contributors [8], [10], [11], [15]. Y-STR analysis can also aid in the identification of haplotypes of missing persons. While the X and Y chromosomes exhibit a degree of homology, they do not undergo genetic recombination during meiosis (except for the pseudoautosomal regions at the chromosome tip) [23]. As a result, most of the Y chromosome is inherited paternally as a block of linked haplotype markers from generation to generation. Thus, males in the same lineage will possess identical Y-STR haplotypes allowing for the determination of a missing person's male haplotype by typing male relatives. Finally, Y-STR analysis can provide additional discriminatory power when combined with (often partial) autosomal STR profile results [24]–[26].

A set of nine Y-STR loci, commonly referred to as the “minimal haplotype loci” set (MHL), have been recommended for use in the forensic community [2]. Subsequently, several additional loci were reported that, in conjunction with the MHL loci, proffered increased discriminatory capacity [9], [27]–[32]. A number of casework-validated commercial Y-STR kits are available and most incorporate 12–17 markers into single multiplex analysis systems [16], [33], [34]. These commercial kits incorporate all twelve of the Y-STR ‘core’ markers that were recommended for forensic use by the US Scientific Working Group on DNA Analysis Methods (SWGDAM) [35]. Despite the robustness of these commercial multiplex Y-STR systems and the ability to discriminate two male individuals in most cases, the coincidence match probabilities between unrelated males are modest compared with the standard set of autosomal STR markers. Hence there is still a need to develop new multiplex systems to supplement these for those cases where additional discriminatory power is desired or where there is a coincidental Y-STR match between potential male participants.

A large number of Y-STR loci (>400) have been identified by various groups and deposited into public databases such as the GDB Human Genome Database (www.gdb.org). A comprehensive annotated STR physical map of the human Y chromosome details the precise location of each locus along the chromosome [36]. Despite the identification and positioning of hundreds of currently known Y-STR loci, a majority of these markers have not been fully characterized with respect to their utility in forensic casework.

A small number of studies have been published involving an in-depth evaluation of a significant numbers of these novel markers [8], [11], [36], [37]. A seminal study by Kayser and co-workers describes an extensive survey of human Y-chromosome microsatellites [37]. While providing an extensive overview, less detail is provided on specific loci, with a small number of loci being identified as the “most variable”. However the gene diversity values used in classifying these loci as variable are based on a population size of only eight male samples. Current forensic literature is replete with small population data studies involving a few novel markers [10], [29], [38]–[49]. However, simply describing individual allele frequencies in numerous populations is not sufficient to determine if a novel Y-STR could be used in forensic casework. While some of these markers may exhibit individually high gene diversity values, few studies have demonstrated the ability of these markers to aid in resolving coincidental matches.

Only a few novel non-‘core’ markers have been incorporated into multiplex PCR systems that have undergone the extensive developmental validation studies [8], [11] required, for example, by US national standards [50]. Without such developmental validation studies it is not possible to evaluate whether the loci are sufficiently robust for use with degraded and limiting quantities of sample in a multiplex analysis format, or can provide sufficient additional discrimination potential when used in combination with other core Y-STR markers.

We have extensively evaluated one hundred and thirty three Y-STR loci (33% of all known loci) for possible use in forensic casework [8]–[11], [36], [42], [43]. Twenty five of these loci were rejected due to poor diversity values or amplification inefficiencies. The remaining one hundred and eight loci have been incorporated into ten multiplex systems that have undergone full developmental validations as required by industry standards ([8], [11], unpublished data). However, due to the limited amount of sample and limited resources of operational crime laboratories, it is unlikely that all ten multiplexes would be employed simultaneously to produce 108-locus profiles. Additionally, not every locus included in these multiplex systems would be beneficial in helping to distinguish between male individuals. Therefore in the present work, attempts were made to construct a non-core loci containing Y-STR multiplex system that, based upon empirical data, offered an extremely high discrimination potential and was robust enough for forensic use.

The result of these efforts was the development of an ultra-high discrimination (UHD) multiplex system incorporating fourteen novel Y-STR loci with a discriminatory capacity greater than that achieved with any commercial kit.

## Methods

### Preparation of Body Fluid Stains

Body fluids were collected from volunteers using procedures approved by the University of Central Florida's Institutional Review Board. Informed written consent was obtained from each donor. Buccal samples were collected from donors using sterile swabs by swabbing the inside of the donor's mouth. Neat semen samples were provided in sealed plastic tubes and stored frozen until they were dried onto sterile cotton swabs. Post-coital cervicovaginal swabs were taken from female participants at various specified time periods subsequent to sexual intercourse. Blood samples were collected by venipuncture and 50 µl aliquots were placed onto cotton cloth and dried at room temperature. Population samples (bloodstains) for gene diversity studies were obtained from the Virginia Division of Forensic Science, Richmond, VA, the South Dakota State Forensic Laboratory, and the Alabama Department of Forensic Sciences. All samples were stored at −47°C until needed.

### DNA Isolation and Purification

DNA was extracted from the buccal swabs, the vaginal swabs, and the semen swabs using a standard phenol: chloroform method [51]. Stains or swabs were cut into small pieces and placed into a Spin-Ease tube (Gibco-BRL, Grand Island NY). The tubes were incubated overnight in a 56°C water bath using 400 µl DNA Extraction Buffer (100 mM NaCl, 10 mM Tris-HCl, pH 8.0, 25 mM EDTA, 0.5% SDS), 0.1 mg/mL Proteinase K, and 10% 0.39 M DTT (added to semen containing samples). After the overnight incubation, swab or stain fabric was placed into a Spin-Ease basket, the basket inserted back into the original tube, and the samples centrifuged at 14,000 rpm for 5 minutes to remove the absorbed fluid from the swab material. A volume of phenol/chloroform/isoamyl alcohol equal to the volume of the crude extract was added and vigorously intermixed by shaking. The aqueous layer, containing the DNA, was removed. Precipitation of the DNA was accomplished by the addition of cold absolute ethanol (two and a half times the volume of the aqueous layer extract) and allowed to progress overnight at −20°C. The DNA was pelleted by centrifugation, washed once using 70% ethanol and re-solubilized with 100 µl of TE^−4^ (10 mM Tris-HCl, 0.1 mM EDTA, pH 7.5) overnight at 56°C.

### Differential Cell Lysis for the Recovery of Sperm DNA

Sperm and non-sperm cells were separated using a standard differential lysis protocol, with minor modifications [52]. Post-coital cervicovaginal swabs were incubated overnight at 37°C in 400 µl of DNA extraction buffer (100 mM NaCl, 10 mM Tris-HCl, 25 mM EDTA, 0.5% SDS) and 0.1 mg/mL Proteinase K. Swab remnants were removed to a Spin-ease basket, the basket inserted back into the original tube, and centrifuged at 14,000 g for 5 min. the resulting supernatant, containing the non-sperm DNA fraction, was removed into a separate tube for further analysis. The sperm pellet was re-suspended in 400 µl of DNA extraction buffer, 0.1 mg/mL Proteinase K, and 40 µl of 0.39 M DTT and incubated for 1 h at 56°C. DNA from both the sperm and non-sperm fractions was isolated and purified using the phenol∶chloroform method described above.

### DNA Isolation and Purification of Dried Blood Samples

The dried bloodstains were incubated overnight at 56°C in 400 µl of DNA extraction buffer (100 mM NaCl, 10 mM Tris-HCl, 25 mM EDTA, 0.5% SDS) and 0.1 mg/mL Proteinase K. The swab pieces were placed into a spin ease basket and centrifuged at 14,000 g for 5 minutes. An equal volume of phenol/chloroform/isoamyl alcohol was added to the crude extract. The aqueous phase extracts containing the DNA were purified using Centricon 100^TM^ concentrators (Millipore, Bedford, MA) according to the manufacturer's instructions.

### DNA Quantitation

DNA was quantitated using ethidium bromide induced fluorescence on a 1% agarose yield gel, using a reference set of DNA standards of known concentration [53].

### Characterization of Genetic Markers

#### Locus Nomenclature

All locus characteristics, including repeat unit structure and size and general chromosome location, were obtained from published sources or by sequencing.

### Multiplex System Development

#### Candidate Y-STR loci

Candidate loci were selected based on diversity value, performance in multiplex systems with forensic samples, and allele size range obtained through previous studies involving developmental validations of novel Y-STR multiplexes [8], [10], [11], [42], [43].

#### Haplotype Diversity

All candidate loci were evaluated individually by determining the contribution each locus would make to increasing the haplotype diversity afforded by the SWGDAM core loci. The HapYDive program was used to determine haplotype diversities (http://www.ipatimup.pt/app/) [54].

### Standard PCR Conditions

#### Reaction Components

Optimization of the UHD multiplex system resulted in a set of standard conditions. The 25 µl reaction mix contained: 1ng of template DNA, 0.08–1.6 µM primers (see below), 1 mM dNTPs, 1X PCR Buffer II (10 mM Tris-HCl, pH 8.3, 50 mM KCl), 1.75 mM MgCl_2_, 10 µg of non-acetylated bovine serum albumin (Sigma-Aldrich, St. Louis, MO), and 2.0 units of AmpliTaq Gold DNA Polymerase (Applied Biosystems).

#### Primers

Primer sequences were obtained from published sources, the Human Genome Database or re-designed using Oligo 6 Primer Analysis Software (Lifescience Software Resource, Long Lake, MN). The primers were tested initially in previously developed multiplexes, first as singleplexes and then in a multiplex system. The primer concentrations for the UHD Multiplex were as follows: DYS444 – 1.04 µM; DYS446 – 0.40 µM; DYS449 – 1.6 µM; DYS459 – 0.28 µM; DYS481 – 0.12 µM; DYS508 – 0.20 µM; DYS522 – 1.6 µM; DYS527 – 1.12 µM; DYS549 – 0.24 µM; DYS552 – 0.56 µM; DYS570 – 0.12 µM; DYS576 – 0.08 µM; DYS607 – 0.24 µM; DYS627 – 1.12 µM; (Invitrogen, Grand Island, NY; Applied Biosystems, Foster City, CA).

#### Cycling Conditions

(1) 95°C 11 min, (2) 32 cycles of 96°C 30 sec, 59°C 1 min 30 sec, 72°C 1 min 30 sec, (3) and final extension 72°C for 45 min.

### PCR Product Detection

A 0.75 µl aliquot of the amplified product was added to 8.7 µl of deionized formamide and 0.3 µl GeneScan 500 LIZ internal lane standard. Tubes containing the above were heated at 95°C for 3 min and snap-cooled on ice for 3 min. Samples were injected onto a Applied Biosystems 3130 Genetic Analyzer, using Module G5 and analyzed with GeneMapper Analysis Software v3.7 using Filter Set G5. A peak detection threshold of 50 RFUs was used for allele designation.

### Multiplex System Performance

#### Multiplex Sensitivity

Different input quantities of template male DNA were tested using the standard UHD multiplex reaction conditions. The amounts tested were: 25 pg, 50 pg, 100 pg, 125 pg, 150 pg, 200 pg, 250 pg, 500 pg, 1 ng, 3 ng, 5 ng, and 10 ng.

#### Specificity

To evaluate possible female DNA cross-reactivity, DNA from female volunteers was tested using the following amounts: 3 ng, 30 ng, 300 ng, and 1 µg.

### Mixture Studies

#### Male/Male

DNA from two individual males was combined in the following ratios: 1/2 (1.5 ng male DNA/1.5 ng male DNA), 1/3 (1.0 ng male DNA/2.0 ng male DNA), 1/6 (0.5 ng male DNA/ 2.5 ng male DNA), 1/12 (0.25 ng male DNA/2.75 ng male DNA), 1/20 (0.15 ng male DNA/2.85 ng male DNA), and 1/30 (0.10 ng male DNA/2.90 ng male DNA). In each case 3 ng of the admixed DNA was tested.

#### Male/Female

3 ng of male DNA was co-amplified with increasing amounts of female DNA in the following ratios: 1/2, 1/10, 1/100, 1/500, 1/1000 and 1/5000.

### Environmentally Impacted Blood Samples

50 µl aliquots of human blood were dried onto cotton cloth. These samples were exposed to different environmental conditions including various temperatures, light sources, and environmental influences including humidity and rain. The environmental conditions were as follows: RTED (room temperature, envelope dried), HLHR (placed outside for exposure to heat, light, humidity, and rain), HLH (placed outside and covered exposure to heat, light, and humidity). Samples from all sets of conditions were collected at varying lengths of time, including 3 days, 1 week, 1 month, 3 months, 6 months, and 1 year. Samples were also exposed to short wave UV at room temperature for 3 days, 1 week, 1 month, 3 months, 6 months and 18 months. For the UV-exposed and room temperature stored samples, 3 ng of input DNA was amplified. For the HLH and HLHR samples, no product was observed during quantitation. Therefore, a 5 µl aliquot of the DNA extract (5%) was used for amplification.

### Mock Casework Samples

#### Post Coital Samples

Post-coital cervicovaginal swabs were taken from a female participant at various time intervals after sexual intercourse (immediately (0 h), 12 h, 24 h, 36 h, and 48 h). Only one set of swabs was taken after each individual act of sexual intercourse to ensure that the amount of semen present at the time was not affected by prior removal of sample. Two swabs were taken at each time interval and DNA isolated as described above. One of the swabs was extracted using a differential extraction method to separate the sperm and non-sperm fractions.

#### Recovery of a Male Y-STR Profile from a Beverage Container

In order to determine if the multiplex could accommodate low copy number forensic samples, swabs of a beverage container lid (plastic coffee cup lid) were collected after being deposited by a male volunteer. The DNA was extracted using the standard phenol:chloroform method described previously. A 5 µl aliquot of the DNA extract (5%) was used for amplification as no product was observed during quantitation.

### Population Studies

#### Descriptive Statistics

The following formulae were used: (1) discriminatory capacity = no. of individuals/no. of different haplotypes observed; (2) gene diversity (*h*), equivalent to the expected frequency of heterozygotes with autosomal diploid loci, was calculated as (n/(n−1))_*_(1−Σp*_i_* ^2^), where p*_i_* = allele frequency at the *i*th locus [55].

## Results

### UHD Loci Selection

In forensic casework, two commercially available Y chromosome STR multiplex amplification kits, the Promega PowerPlex® Y kit and the Applied Biosystems AmpF*l*STR® Yfiler® kit, are widely used. These commercial kits employ 12 and 17 markers respectively, and both include the SWGDAM core loci. While the kits have the ability to distinguish most male individuals in a particular case, the occurrence of coincidental matches between two unrelated individuals can still occur. A number of other non-core Y-STR loci have been identified on the Y chromosome that, hypothetically, could provide the ability to resolve a majority of these coincidental matches. We previously evaluated and characterized 133 of the ∼400 known Y-STR loci (33%). This resulted in the development of ten novel Y-STR multiplex systems encompassing 91 non-core Y-STR loci in addition to the loci contained in the commercial kits [8]–[11], [42], [43]. The design and developmental validation of the these multiplex systems resulted in a determination of each locus' performance within a multiplex system format [8], [11] as well as its gene diversity [8], [10], [11], [42], [43].

Initial UHD design specifications were: (i) that the number of loci would not exceed 15 since our previous experience with multiplex development indicated that the most robust systems contain 15 or fewer loci (data not shown), and (ii) that only loci that were not already incorporated into commercial kits were considered. A number of non core candidate loci were rejected as UHD candidates due to performance limitations, low or inaccurate (due to sampling effects) gene diversity values and this resulted in a ‘first pass’ candidate list of 65 loci. The candidate loci and their associated gene diversities are listed in Table 1.

**Table 1:** Diversity Value for UHD Candidate Loci.

Locus	MP location	Diversity Value (Combined)
DYS464	MP4	0.99
DYS527	MP4	0.92
DYS627	MP8	0.86
DYS481	MP8	0.84
DYS449	MP4	0.84
DYS576	MP5	0.83
DYS557	MP4	0.79
DYS570	MP5	0.79
DYS643	MP6	0.78
DYS447	MP4	0.77
DYS607	MP5	0.77
DYS459	MP5	0.75
C4	MP2	0.74
DYS448	MP4	0.73
DYS463	MP4	0.73
DYS508	MP6	0.73
DYS446	MP3	0.72
DYS549	MP5	0.72
A7.1	MP2	0.71
DYS443	MP4	0.71
A10	MP3	0.70
DYS598	MP5	0.69
DYS441	MP3	0.68
DYS505	MP6	0.68
DYS456	MP4	0.67
DYS468	MP4	0.66
DYS485	MP6	0.66
DYS522	MP4	0.64
DYS452	MP4	0.64
A7.2	MP1	0.63
DYS442	MP3	0.62
DYS484	MP4	0.62
DYS533	MP6	0.62
DYS437	MP2	0.61
DYS495	MP6	0.61
DYS444	MP6	0.61
DYS513	MP5	0.60
H4	MP2	0.59
DYS455	MP4	0.59
DYS552	MP6	0.58
DYS556	MP6	0.57
DYS594	MP5	0.56
DYS531	MP4	0.55
DYS462	MP3	0.54
DYS561	MP5	0.52
DYS494	MP6	0.51
YAP	MP3	0.50
DYS426	MP3	0.48
DYS578	MP6	0.46
DYS445	MP4	0.41
DYS588	MP4	0.41
DYS453	MP4	0.40
DYS540	MP6	0.38
DYS388	MP2	0.33
DYS638	MP6	0.28
DYS488	MP5	0.23
DYS454	MP4	0.19
DYS641	MP6	0.16
DYS434	MP2	0.12
DYS476	MP5	0.12
DYS425	MP2	0.11
DYS590	MP5	0.09
DYS436	MP3	0.08
DYS435	MP3	0.05
DYS575	MP5	0.05

The forensic usefulness of a Y-STR locus in distinguishing individuals is not solely determined by its gene diversity values but is also related to how well it complements the other markers in the typing set used. A person's Y-STR haplotype consists of a set of physically and genetically linked STR markers that are co-typed subsequent to multiplex amplification with a standard set of well characterized core loci. The extent to which an additional locus adds to the discriminatory capacity of the multiplex system depends not only upon the inherent genetic diversity of the locus (the number and frequencies of alleles) but also upon the evolutionary history of the locus in question within different haplogroups. A multi-locus Y-STR haplotype will often be very predictive of (or even define) the Y-SNP haplogroup to which an individual belongs and thus the utility of adding another Y-STR marker to the typing set depends upon the extent to which it is able to further differentiate individuals within that particular haplogroup. Thus a locus displaying low variance in allele frequencies within the particular haplogroup would be less useful in discriminating individuals than one that had a high variance. It is thus conceivable that a particular Y-STR locus that demonstrates high gene diversity in the general population (that comprises individuals belonging to multiple haplogroups) would not add, due to its low intra-haplogroup variance, a significant amount of additional discrimination power to the multiplex.

**Figure 1:**
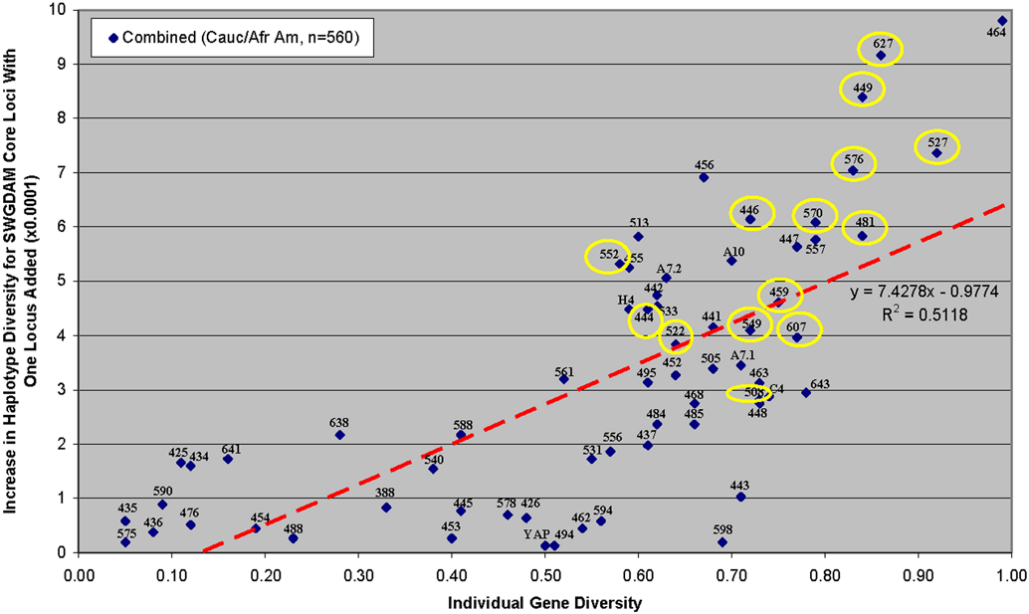
Increase in haplotype diversity for SWGDAM core loci with additional locus added. The 14 UHD loci are circled.

In order to incorporate the most informative loci from the non core 65 candidates into the UHD multiplex, the relative ability of each of the candidates in improving the discriminatory power of the core loci set was empirically determined. For this, a large mixed African American and Caucasian population (n = 560) expected to contain most of the major haplogroup classes present in these two populations was used. Firstly the haplotype diversity of the SWGDAM core loci was determined. Then each of the 65 candidate loci was added to the SWGDAM core set and the increase in haplotype diversity determined. For each locus, the single locus diversity value was plotted against the increase in multi-locus haplotype diversity achieved by that locus when used in combination with the SWGDAM loci (Figure 1). In general there was a correlation between the single locus diversity and its ability to enhance the multi-locus diversity of the SWGDAM multiplex (r^2^ = 0.51). Loci with low individual diversity values (e.g. DYS425 (0.11); DYS434 (0.12); DYS435 (0.05); DYS436 (0.08); DYS476 (0.12); DYS575 (0.05); DYS590 (0.09); DYS641 (0.16)) produced a small increase in multi-locus haplotype diversity for the SWGDAM core loci. Many of the loci with high diversity values (e.g. DYS449 (0.81); DYS456 (0.67); DYS464 (0.99); DYS527 (0.92); DYS570 (0.79); DYS576 (0.83); DYS627 (0.86) resulted in a larger increase in haplotype diversity. However several of the loci resulted in little or no increase in haplotype diversity, despite a moderate diversity value (DYS426-0.48; DYS445-0.41; DYS453-0.40; DYS462-0.54; DYS494-0.51; DYS578-0.46; DYS594-0.56; DYS598-0.59; YAP-0.50) and illustrates the need for empirical testing of the relative efficacy of loci for complementing a set of multiplexed loci.

Loci exhibiting a high diversity value and a large increase in haplotype diversity (upper right portion of the plot above the regression line)) were considered for inclusion into the UHD multiplex. Multi-copy loci with >2 copies were excluded due to complications with their analysis. Based on compatible allele size ranges, the availability of dye labels, and annealing temperatures, fourteen novel Y-STR loci were selected for inclusion into the UHD multiplex and: DYS444, DYS446, DYS449, DYS459, DYS481, DYS508, DYS522, DYS527, DYS549, DYS552, DYS570, DYS576, DYS607, and DYS627 (Figure 1). Loci characteristics (repeat motif, size range, number of alleles, average percent stutter, and primer sequences) are provided in Table 2.

**Table 2:** UHD Loci Characteristics.

Locus	GDB Accession Number	Repeat Motif	Alleles	Size Range (bp)	Avg % Stutter	Primer Sequences With Labled Dyes
**DYS481** [**^43^**]	11503780	CTT	18–31	115–158	18.8	FF: AGGAATGTGGCTAACGCTGT
						R: ACAGCTCACCAGAAGGTTGC
**DYS576** [**^11^**]	11503970	(AAAG)_n_	12–23	167–209	9.0	FF: TTGGGCTGAGGAGTTCAATC
						R: GGCAGTCTCATTTCCTGGAG
**DYS570** [**^11^**]	11503958	(TTTC)_n_	13–22	244–280	9.2	FF: GAACTGTCTACAATGGCTCACG
						R: TCAGCATAGTCAAGAAACCAGACA
**DYS527** **[^10]^**, [11]	11503872	(GAAA)_4_(AGAA)_1_(GGAA)_3_(ATGA)_1_(AACA)_1_(AGAA)_1_(AGGA)_1_(AAGA)_14_(AAGG)_n_(AAAG)	28–38	326–365	7.2	FF: TCGCAAACATAGCACTTCAG
						R: TTCTAGGAAGATTAGCCACAACA
**DYS459** [**^11^**]	11498133	(ATTT)_n_	6–10	136–156	4.1	FV: CAGGTGAACTGGGGTAAATAAT
						R: TTGAGCAACAGAGCAAGACTTA
**DYS549** [**^11^**]	11503916	(GATA)n	8–15	220–250	5.8	FV: AACCAAATTCAGGGATGTACTGA R: GTCCCCTTTTCCATTTGTGA
**DYS444** [**^43^**]	10807128	(ATAG)_n_	10–15	291–311	4.2	FV: TCTAAGGGATCCAAAGGCAGAA
						R: GTGTGAACCATTTGGCATGTTTA
**DYS449** [**^10]^**, [11]	10879367	(TTTC)_n_N_50_(TTTC)_n_	25–37	344–392	10.0	FV: TGGAGTCTCTCAAGCCTGTTCTA
						R: CCTGGAAGTGGAGTTTGCTGT
**DYS508** [**^43^**]	11503834	(TATC)_n_	9–15	170–195	5.2	FN: ACAATGGCAATCCCAAATTC
						R: GAACAAATAAGGTGGGATGGAT
**DYS552** [**^43^**]	11503922	(TCTA)_3_(TCTG)_1_(TCTA)_n_N_40_(TCTA)_n_	21–27	231–257	7.5	FN: CCATAGTCCGAGGTCAAGT
						R: AACACCTGATGCCTGGTTG
**DYS522** [**^10]^**, [11]	11503862	(GATA)_n_	8–14	344–347	4.1	FN: CCTTTGAAATCATTCATAATGC
						R: TCATAAACAGAGGGTTCTGG
**DYS446** [**^11^**]	10873760	(TCTCT)_n_	10–23	95–160	6.5	FP: TATTTTCAGTCTTGTCCTGTC
						R: GAGACTCTGTCTGAAGAGAG
**DYS607** [**^11^**]	11505463	(GAAG)_n_(GAAA)_1_(GAAG)_1_(GAAA)_1_(GAAG)_1_	10–17	180–208	10.6	FP: AGCATACAGCGTAATCACAGC
						R: TCAGACAAAGCCCAGTTGAG
**DYS627** [**^43^**]	11510455	(AAAG)_3_(GAAA)_1_(AAAG)_3_(AGAG)_2_(AGAA)_n_(AAAG)_1_(AGAA)_1_	49–61	302–350	8.1	FP: CTAGGTGACAGCGCAGGATT
						R: GGATAATGAGCAAATGGCAAG

F = FAM; V = VIC; N = NED; P = PET.

### UHD Performance Characteristics

#### PCR Optimization

Multiplex PCR reaction conditions were optimized by altering the concentration of critical PCR reagents and the thermocycling conditions and using both male (3 ng) and female (300 ng) DNA as input template. This resulted in the determination of a set of standard reaction conditions described in the Methods Section.

#### Sensitivity

**Figure 2:**
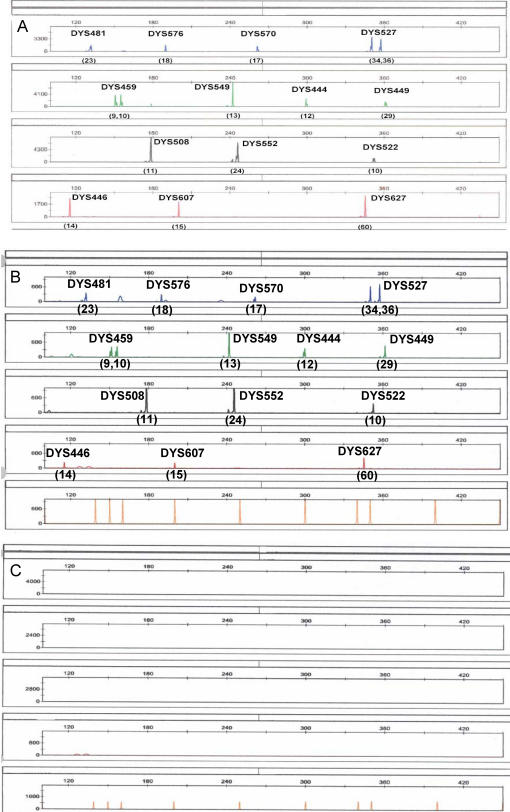
Sensitivity and specificity of the UHD multiplex. A) Full 14-locus profile obtained with a standard input of 500 pg of male template DNA; B) Profile obtained with 25 pg of male template DNA; C) lack of products with 1 µg of female template DNA.

A forensic DNA typing system needs to be able to work with sub-nanogram quantities of template DNA. The sensitivity of the UHD multiplex was tested using varying amounts of input template male DNA (25 pg–10 ng). Although 500 pg (Figure 2A) to 2 ng of DNA gave optimal signal intensity and inter-locus balance, a full 14-locus male Y-STR haplotype was still obtained with 25 pg of DNA (Figure 2B), which is equivalent to ∼4–5 diploid cells.

#### Specificity

A significant benefit of using Y-STR analysis is the specific amplification of male DNA in male/female DNA admixtures, a situation that arises often in biological stains recovered from sexual assault investigations. Although there is no genetic recombination between the X and Y chromosomes, the presence of homologous sequences on the X chromosome could confound Y-STR analysis by titrating out critical reagents or producing pseudo-Y alleles. Thus empirical testing of Y-STR primer sets for female DNA cross reactivity is essential. The UHD was tested with varying amounts of female DNA (3 ng–1 µg) and no significant amplification products were observed (Figure 2C).

#### Mixtures

##### Male/Male

Often in forensic casework there is more than one male contributor to a sample. With standard autosomal STR analysis is often difficult to determine the number of contributors in an admixture. Due to the hemizygous nature of Y-STR loci, with only one allele being found at most loci, determination of the number of male contributors is facile.

**Figure 3:**
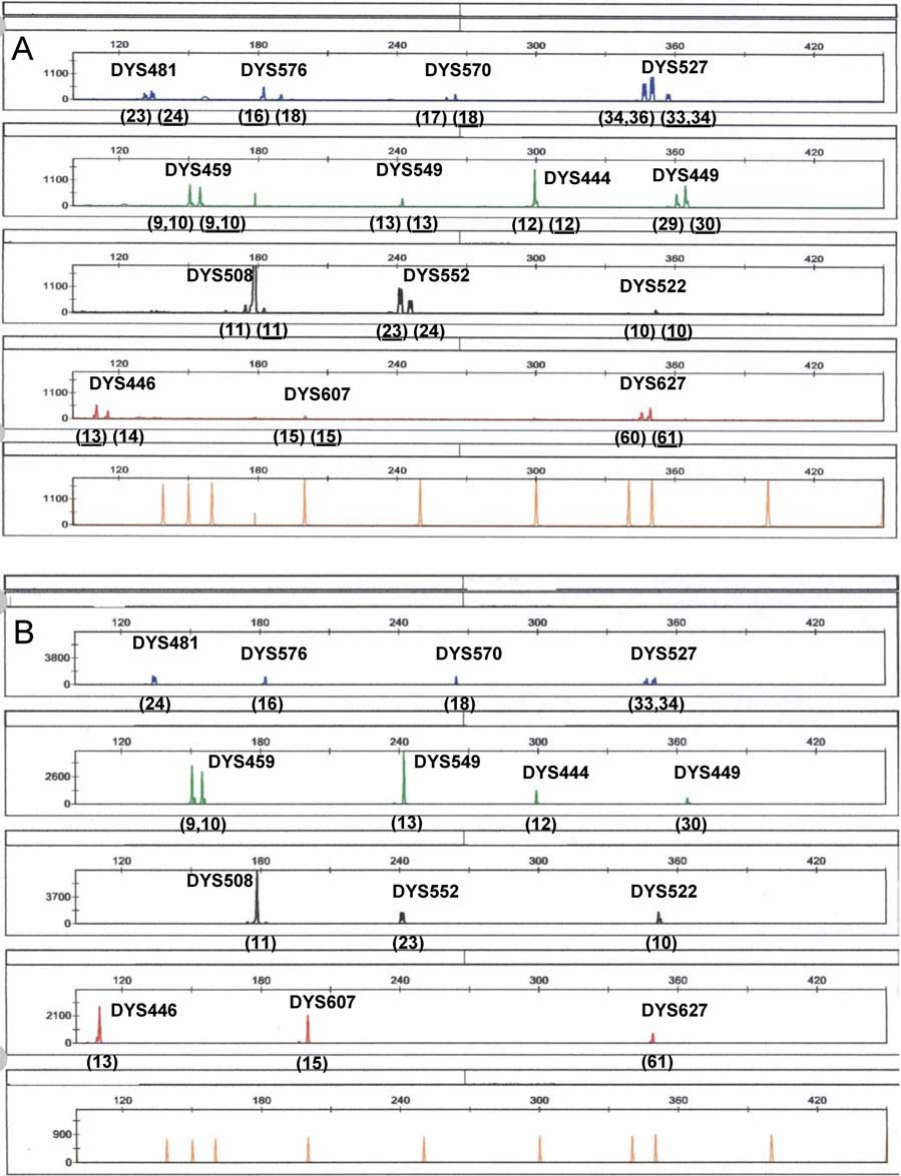
Mixtures. A) Male/Male Admixed DNA sample. Full UHD profile of major and minor components obtained with minor component comprising 1/6^th^ of input DNA. Donor haplotypes provided in parentheses below each locus. Minor component alleles are underlined. B) Male/Female Mixture - Male/Female Admixed DNA sample. Full UHD profile obtained at a male/female DNA ratio of 1/5000. Male donor alleles indicated in parentheses below each locus.

In order to evaluate the ability of the UHD multiplex to identify multiple male contributors, male/male DNA admixtures from two unrelated individuals were tested at various ratios including 1/2, 1/3, 1/6, 1/12, 1/20 and 1/30. For each admixture, 3 ng of total DNA was amplified. A full minor male component profile was obtained when it comprised 1/2, 1/3 and 1/6 of the total male DNA (Figure 3A). Partial minor male component profiles were obtained when it comprised 1/12 and 1/20 of the total DNA (data not shown).

##### Male/Female

To test the ability of the UHD multiplex to produce a male haplotype in the presence of large quantities of female DNA, a situation that is frequently encountered in actual casework, a series of male/female admixtures were prepared in which 3 ng of total male DNA was amplified, with varying quantities of female DNA. Full 14-locus profiles were obtained when the male DNA component comprised 1/2, 1/10, 1/100, 1/500 1/1000, and 1/5000 (Figure 3B) of the total DNA.

#### Environmental Effects

For a multiplex system to be useful in forensic casework, it must permit the recovery of DNA profiles from samples that have been exposed to various environmental conditions. To assess the ability of the UHD multiplex to recover Y-STR haplotypes from environmentally compromised samples, bloodstains originating from the same male individual were exposed to different environmental conditions including various temperatures, light sources, and environmental influences including humidity and rain. For this study, samples were grouped into four categories: 1) dried blood stains stored in envelopes at room temperature (RTED), 2) dried blood stains left outside and uncovered (HLHR), exposed to heat, light, humidity, and rain; 3) dried blood stains exposed to UV light (UV); and 4) dried blood stains left outside and covered (HLH), exposed to heat, light, and humidity. Samples were left exposed to these conditions and collected at various time intervals including three days, one week, one month, three months, six months, one year and, for the UV exposure set, eighteen months.

**Figure 4:**
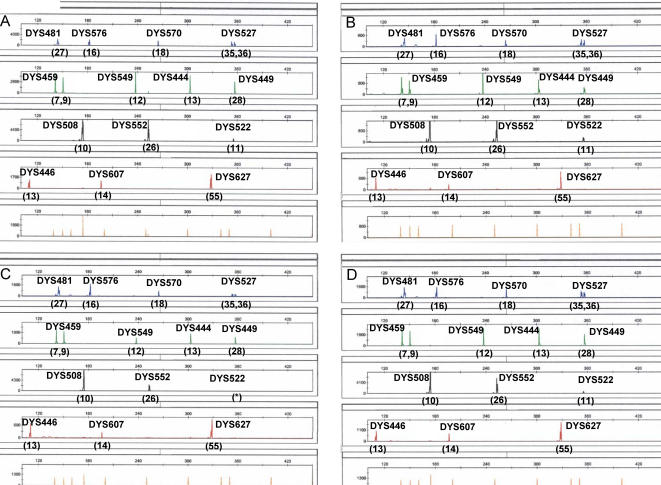
Stability of environmentally compromised blood samples. Full fourteen-locus haplotypes for the UHD multiplex obtained from bloodstains that had been exposed to A) room temperature storage for 1 year; B) UV light for 1.5 years; C) heat, light, humidity, and rain for 1 month; D) heat, light, and humidity for 1 month.

For the RTED samples, full profiles were recovered for all samples up to and including one year, with no observed reduction in allelic signal intensities as the time interval increased (Figure 4A). Full profiles were also obtained from the UV exposed samples, up to and including eighteen months (Figure 4B).

The HLHR and HLH samples were placed in an outdoor, un-wooded area in Orlando, Florida which has a semi-tropical climate. During the course of this study (one year), these samples were exposed to temperatures ranging from 32°F to 94°F (average high of 88°F and average low of 37°C) and the HLHR samples were additionally exposed to rain fall on 137 days out of the year (the three-day samples received one day of rain; the one-week samples received one day of rain; the six-month samples received 73 days of rain; and the one year sample received 137 days of rain). Complete 14-locus profiles were obtained from the one month exposed HLHR (Figure 4C) and HLH (Figure 4D) samples. These latter somewhat-unexpected results were encouraging from the casework standpoint, since heat, light, humidity and rain are often detrimental to DNA analysis.

#### Mock Casework Samples

##### Post-Coital Cervicovaginal Samples

**Figure 5:**
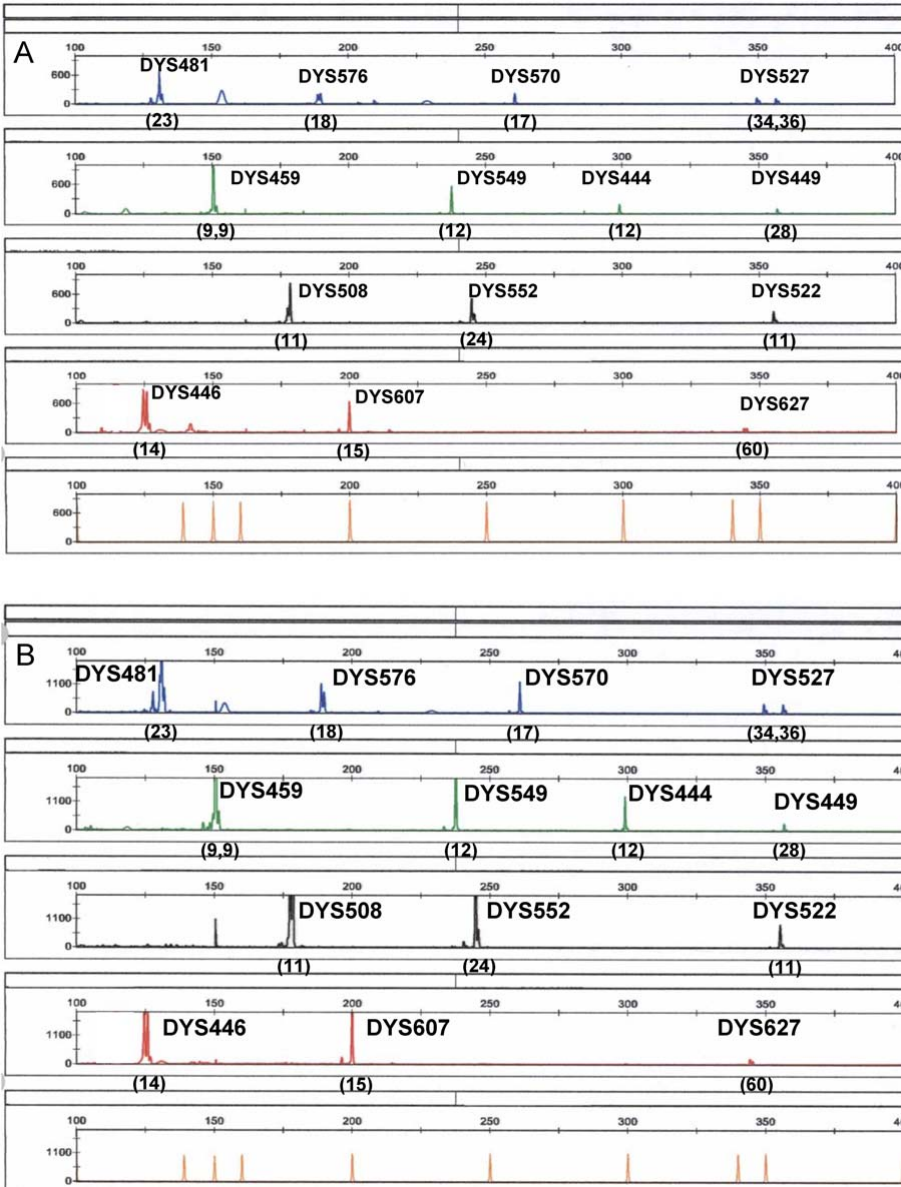
Post-coital cervicovaginal samples. Complete UHD profiles obtained from 3 ng of the sperm fraction DNA recovered 48 hrs after intercourse (A), and 100 ng of a non-differential (i.e. sperm and non-sperm) DNA fraction recovered 48 hrs after intercourse (B).

A series of post-coital cervicovaginal samples were collected from a female donor 0 to 48 h (in 12 h intervals) after intercourse. Each time point sample was collected after a separate act of sexual intercourse and was preceded by a five day abstention period. As a negative control, a pre-coital swab was also recovered prior to coitus for each sampling and only data from post-coital samples that demonstrated a lack of male DNA in the associated pre-coital sample was used. The participant was instructed to carry out normal daily functions to permit the normal loss of semen constituents experienced over time and that often confounds the analysis of *bona fide* casework samples. Using a differential lysis DNA isolation method, the ‘sperm’ and ‘non-sperm’ DNA fractions were separated. Approximately 3 ng of the sperm or 100 ng of the non-sperm DNA fractions was analyzed by the UHD system. Complete 14-locus Y-STR profiles were recovered from the 0, 12, 24, and 48 h post coital samples from the sperm fractions (Figure 5A), all of which were consistent with the semen donor's known profile.

In a differential lysis procedure, sperm from low copy number samples (i.e. extended interval post-coital samples) experience varying degrees of loss due to the inherent limitations of the physical manipulations required to carry out the differential extraction protocol. Therefore, a standard ‘non-differential’ DNA extraction was performed on the second swab for each time interval thus eliminating the need for separation, and potential loss, of the sperm component. Again, complete male profiles consistent with the known donor were obtained from the 0, 12, 24 and 48 hour samples (Figure 5B).

##### Recovery of a Male Y-STR Profile from a Beverage Container Lid

**Figure 6:**
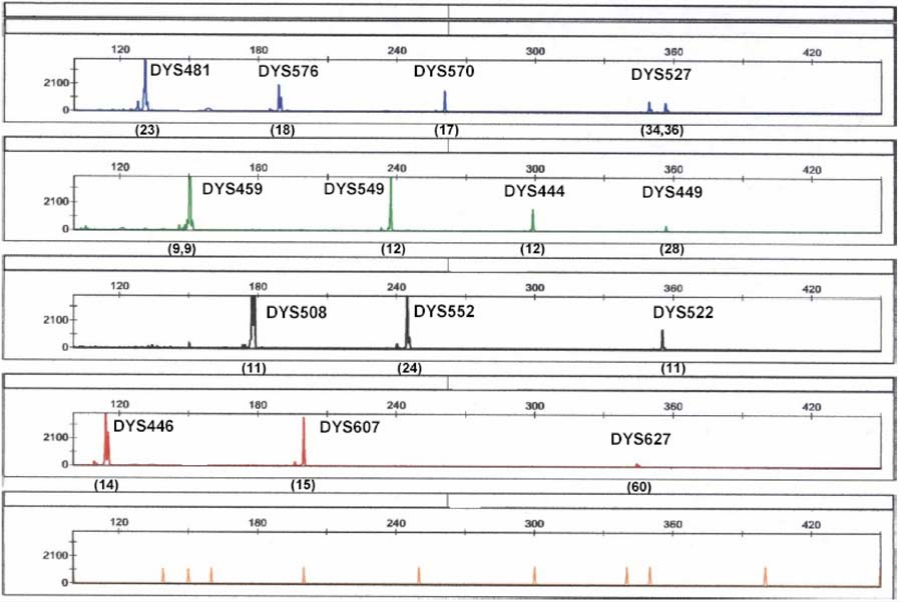
Mock casework samples. Full UHD profile obtained from male DNA recovered from a beverage container lid.

Limited amounts of DNA are often recovered from evidentiary materials. A male participant provided a beverage container lid from which DNA was successfully recovered. A complete fourteen-locus UHD Y-STR profile consistent with the known donor was recovered from the beverage container lid (Figure 6).

### Efficacy of the UHD Multiplex for Differentiating Individuals

#### Multi-Locus Discrimination Potential

We specifically incorporated into the UHD a number of loci that, individually, provided enhanced discriminatory power to the SWGDAM core set (Figure 1). In order to empirically determine the discriminatory capacity of the UHD system as a whole a population study was carried out. Fourteen-locus UHD haplotypes were obtained from 191 Caucasian American and 381 African American individuals and the number of unique and matching multi-locus haplotypes determined (Table 3). Among Caucasian American samples, 190 unique haplotypes were observed, and among the 381 African American samples, 380 unique haplotypes were observed (Table 3) resulting in a discriminatory capacity of 99.5% and 99.7% respectively. The discrimination capacity was 99.7% when the population samples were combined (570 unique haplotypes/572 individuals). Additional typing using non-UHD loci indicated that the multilocus haplotype shared between one pair of Caucasian American individuals and the (different) multilocus haplotype shared between one pair of African American individuals were *bona fide* coincidental matches between unrelated individuals (data not shown).

**Table 3:** UHD Multiplex Discrimination Compared with Commercial Kits.

UHD	Caucasians	African Americans	Total
**Number of Individuals**	191	381	572
**Number of Haplotypes**	190	380	570
**Discriminatory Capacity**	99.5%	99.7%	99.7%
**No. of Haplotypes Observed Only Once (%)**	189 (99.0%)	379 (99.5%)	568 (99.3%)
**Occurrence of Most Frequent Haplotype (%)**	2 (1.0%)	2 (0.5%)	2 (0.3%)
ABI AmpF*l*STR® Yfiler®	Caucasians	African Americans	Total
**Number of Individuals**	191	381	572
**Number of Haplotypes**	190	378	563
**Discriminatory Capacity**	99.5%	99.2%	98.4%
**No. of Haplotypes Observed Only Once (%)**	189 (99.0%)	375 (98.4)	555 (97.0%)
**Occurrence of Most Frequent Haplotype (%)**	2 (1.0%)	2 (0.5%)	3 (0.5%)
Promega PowerPlex® Y	Caucasians	African Americans	Total

The same population sample set was used to determine the discriminatory power afforded by the two most discriminatory commercial Y-STR multiplex systems, the Promega PowerPlex® Y kit and the Applied Biosystems AmpF*l*STR® Yfiler® kit (Table 3) [33], [34]. UHD was found to possess a higher discriminatory capacity than either commercial kit. Among the Caucasian American samples, 152 unique PowerPlex® Y and 189 unique Yfiler® haplotypes were present compared with 189 unique UHD haplotypes. Among the African American samples, 328 unique PowerPlex® Y and 375 unique Yfiler® haplotypes were present compared with 379 unique UHD haplotypes. Amongst the 572 combined population samples included in this study, 500 unique haplotypes were obtained using the Promega PowerPlex® Y kit, resulting in an overall multiplex discriminatory potential of 87.4%. Only 462 haplotypes (80.8%) were observed once in the data set and the most frequent haplotype was observed ten times. Using the Applied Biosystems AmpF*l*STR® Yfiler® kit, 563 unique haplotypes were obtained, resulting in an overall multiplex discriminatory potential of 98.4%, an 11% increase compared to the PowerPlex® Y system. However, using the UHD multiplex, 570 unique haplotypes were obtained, resulting in the highest discrimination potential (99.7%) of the multiplexes compared in this study (∼12% increase compared to PowerPlex® Y; ∼1% increase compared to AmpF*l*STR® Yfiler®).

#### UHD Augmentation of Commercial Kits

The UHD was designed to enhance the discrimination afforded by the commercially available Y-STR multiplex systems. The augmentation efficacy was evaluated by determining the extent to which the UHD loci would discriminate individuals who would otherwise be indistinguishable with the commercial kits.

For the PowerPlex® Y system, 110 of the 572 (∼19%) samples did not possess a unique haplotype. These 110 individuals represented 38 distinct haplotypes, comprising 2–10 individuals per haplotype group (Table 4). All of the individuals within these 38 haplogroup classes, except for one pair, were distinguishable by the UHD system (Table 4). Thus, only two individuals (0.3%) did not possess a unique PowerPlex® Y+UHD combined haplotype, a significant reduction from the 19% with PowerPlex® Y alone.

**Table 4:** Resolution of Coincidental Matches with the Promega PowerPlex® Y kit.

N = 572	No. of Samples With Same Power Plex® Y Profile	No. of Samples With Same Power Plex® Y+UHD Profile
Haplotype 1	2	0
Haplotype 2	2	0
Haplotype 3	2	0
Haplotype 4	6	0
Haplotype 5	2	0
Haplotype 6	4	0
Haplotype 7	3	0
Haplotype 8	3	0
Haplotype 9	10	2
Haplotype 10	5	0
Haplotype 11	2	0
Haplotype 12	2	0
Haplotype 13	8	0
Haplotype 14	2	0
Haplotype 15	2	0
Haplotype 16	3	0
Haplotype 17	2	0
Haplotype 18	2	0
Haplotype 19	2	0
Haplotype 20	6	0
Haplotype 21	2	0
Haplotype 22	3	0
Haplotype 23	2	0
Haplotype 24	2	0
Haplotype 25	2	0
Haplotype 26	2	0
Haplotype 27	2	0
Haplotype 28	2	0
Haplotype 29	3	0
Haplotype 30	4	0
Haplotype 31	2	0
Haplotype 32	2	0
Haplotype 33	2	0
Haplotype 34	2	0
Haplotype 35	2	0
Haplotype 36	2	0
Haplotype 37	2	0
Haplotype 38	2	0
**Total**	**110**	**2**

For the more discriminating AmpF*l*STR® Yfiler® system, 17 of the 572 (∼3%) samples did not possess a unique haplotype. These individuals comprised 8 different haplotype groups that consisted of 2 to 3 individuals (Table 5). With the UHD all samples could be distinguished. Thus, the combination of the AmpF*l*STR® Yfiler® system and the UHD multiplex was able to distinguish all 572 individuals in the sample set.

**Table 5:** Resolution of Coincidental Matches with the Applied Biosystems AmpF*l*STR® Yfiler® kit.

N = 572	No. of Samples With Same Yfiler® Profile	No. of Samples With Same Yfiler®+UHD Profile
Haplotype 1	2	0
Haplotype 2	2	0
Haplotype 3	2	0
Haplotype 4	2	0
Haplotype 5	3	0
Haplotype 6	2	0
Haplotype 7	2	0
Haplotype 8	2	0
**Total**	**17**	**0**

## Discussion

In this work we designed and developed a highly discriminating robust multiplex Y-STR typing system that uses loci that are not present in any of the commercially available Y-STR systems. Rationalization of the loci selection process means that, at least for the American-Caucasian and African-American populations, it is probably the most discriminating Y-STR system available at this time. It is principally intended for forensic casework use but could have a number of other applications, particularly in the field of human evolutionary genetics and genetic genealogy. It can be used either as a stand alone system in its own right or to augment the discriminatory power of the commercially available Y-STR multiplex systems.

Candidate loci were selected based upon empirical studies that evaluated the relative ability of each locus to augment the discriminatory potential of a set of routinely used core loci. Due to genetic admixture within the major bio-geographic-specific haplogroup sub-populations, a high single locus Y-STR haplotype diversity calculated from a mixed ‘total’ population does not necessarily translate into an equally high increase in discrimination when combined with the core loci. However we found that, in general, there was a correlation between the single locus diversity and its ability to enhance the multi-locus diversity of a core loci multiplex (r^2^ = 0.51). Nevertheless we rejected a number of candidates that, despite possessing a reasonably high diversity, provided weak augmentation power (e.g. DYS594, DYS 598).

The result of the extensive evaluation of the non-core loci was the development of an ultra high discrimination (UHD) multiplex that permits the robust co-amplification of 14 non-core Y-STR loci. Analysis of 587 Caucasian American and African American samples produced an overall multiplex discriminatory potential of 99.7%. In comparison, the two commercially available Y-STR multiplex kits, Promega's PowerPlex® Y and Applied Biosystems' AmpF*l*STR® Yfiler®, produced discriminatory capacities of only 87.4% and 98.4%, respectively. Thus the ability of the UHD multiplex to distinguish individual male samples surpasses that of the two commercially available multiplex systems currently used in forensic casework. Additionally, the use of the UHD multiplex in conjunction with Applied Biosystems' AmpF*l*STR® Yfiler® system resulted in the ability to distinguish all 587 samples.

The UHD multiplex has been evaluated for use with samples frequently encountered in forensic casework. Based upon a number of studies including specificity, sensitivity, discriminating capacity and performance with non-probative casework specimens, the system has demonstrated its potential forensic casework utility to augment the core loci contained in commercially available kits.
